# Neural dynamics based on the recognition of neural fingerprints

**DOI:** 10.3389/fncom.2015.00033

**Published:** 2015-03-24

**Authors:** José Luis Carrillo-Medina, Roberto Latorre

**Affiliations:** ^1^Departamento de Eléctrica y Electrónica, Universidad de las Fuerzas Armadas ESPESangolquí, Ecuador; ^2^Universidad Autónoma de MadridMadrid, Spain

**Keywords:** neuron signature, local contextualization, local discrimination, processing based on signal discrimination, multicoding, self-organizing neural network

## Abstract

Experimental evidence has revealed the existence of characteristic spiking features in different neural signals, e.g., *individual neural signatures* identifying the emitter or *functional signatures* characterizing specific tasks. These *neural fingerprints* may play a critical role in neural information processing, since they allow receptors to discriminate or contextualize incoming stimuli. This could be a powerful strategy for neural systems that greatly enhances the encoding and processing capacity of these networks. Nevertheless, the study of information processing based on the identification of specific neural fingerprints has attracted little attention. In this work, we study (i) the emerging collective dynamics of a network of neurons that communicate with each other by exchange of neural fingerprints and (ii) the influence of the network topology on the self-organizing properties within the network. Complex collective dynamics emerge in the network in the presence of stimuli. Predefined inputs, i.e., specific neural fingerprints, are detected and encoded into coexisting patterns of activity that propagate throughout the network with different spatial organization. The patterns evoked by a stimulus can survive after the stimulation is over, which provides memory mechanisms to the network. The results presented in this paper suggest that neural information processing based on neural fingerprints can be a plausible, flexible, and powerful strategy.

## 1. Introduction

*Intraburst neural signatures* were first described for the neurons of the pyloric central pattern generator (CPG) of the lobster stomatogastric nervous system (Szücs et al., 2003, 2005). They consist of very precise spike timings in the bursting activity of some cell-types. Recent experimental findings in this circuit demonstrate that the reproducibility of these *neural fingerprints* allows us to identify the source of signals with different bursting frequencies and number of spikes per burst, even across different species (Brochini et al., 2011). The existence of intraburst neural signatures has also been reported in other living neurons, such as subthalamic neurons (Garcia et al., 2005), mammalian retinal ganglion cells (Zeck and Masland, 2007) or leech heartbeat CPG motoneurons (Campos et al., 2007). Similarly, other characteristic stereotyped firing patterns have been discovered in other neural circuits. For example, experimental evidence shows that some neural systems can exhibit *functional or behavioral neural signatures* representing different states or associated to the task performed at a given moment (Klausberger et al., 2003; Somogyi and Klausberger, 2005; Kaping et al., 2011). The observation of different neural fingerprints in widely different vertebrate and invertebrate neural systems suggests that they can have important functional implications for the circuit where they are present. In this vein, model circuits inspired by the pyloric CPG point out that the characteristic collective behavior of this neural circuit can drastically change when the intraburst neural signature of some of its neurons is modified (Latorre et al., 2002; Rodríguez et al., 2002; Latorre et al., 2004).

Neural signatures characterizing specific signals can be a mechanism used by the nervous system to contextualize or discriminate neural information. Information processing based on the recognition of these neural fingerprints can make use of this recognition, for example, to decide or weight the decision about the output of a neuron, or to emit a new neural fingerprint in the output. Theoretical efforts can largely help to address the information processing based on the emission and recognition of neural fingerprints. Beyond the context of CPG circuits, to our knowledge, only a binary model has been proposed with this goal (Tristán et al., 2004). Additionally, multicoding strategies including intraburst neural signatures have been recently investigated in the context of a spiking neural network (Latorre, in revision). In the present work, we use the same simple approach as Tristán et al. (2004) to study how a large-scale network can detect and discriminate specific neural fingerprints (which can be associated to particular spiking features of other neural areas) in its external stimuli and encode this input in its collective dynamics. The goal is to assess the viability of a neural processing strategy using neural fingerprints, as well as to investigate the underlying encoding mechanisms arising in the network. We are particularly interested in the influence of the network topology on the properties of the dynamic organization of the fingerprint-based dynamics. In the existing models (Tristán et al., 2004; Latorre, in revision), only a regular topology has been used as connection pattern in the network. Here, we use a small-world topology, i.e., networks whose units are organized in densely linked groups that are sparsely but reciprocally interconnected. This pattern of connectivity can provide relevant computational properties to the network (Lago-Fernández et al., 2000; Latora and Marchiori, 2001). All the source code used in the simulations reported in this paper can be found in http://www.ii.uam.es/~rlatorre/source_code.tgz.

The ability of the network to encode and process information is related to the detection and propagation of specific stimuli. We first carry out an analysis of autonomous networks and study the self-organizing properties within networks receiving a single external stimulus. Then, we compare the collective dynamics of networks where multiple stimuli are introduced both in series and in parallel. Simulations reveal that the network displays complex self-organizing properties. Fast transitions of the collective activity emerge in response to the arrival of specific neural fingerprints as external input. These responses are organized as localized patterns of activity with different spatial organization that coexist and compete in the network. The parameters that define the subcellular information processing and the specific organization of the connections among neurons tune the self-organizing properties of the network and have a strong influence on its ability to sustain different stimuli. These factors provide short-term and long-term memory mechanisms to store incoming stimuli after the stimulation is over.

## 2. Materials and methods

### 2.1. Single neuron model

**Figure 1:**
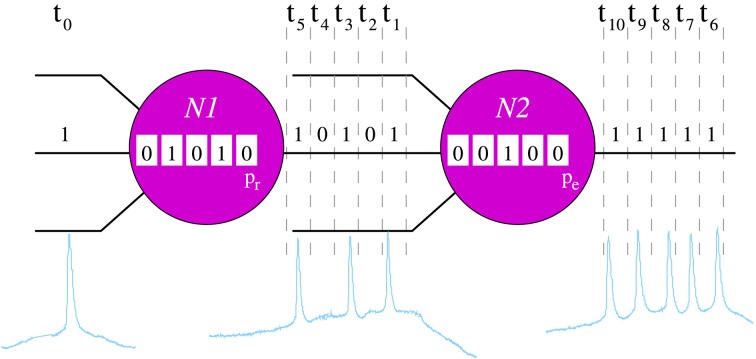
**Schematic representation of the processing rules in the neuron model**. For the sake of simplicity, in this example we only consider an input channel and its corresponding local informational context (sequence of 5 bits represented inside each neuron). In the example, neurons recognize a unique 5-bit neural fingerprint, *F* = (1, 0, 1, 0, 1), and the spontaneous binary pattern is (1, 1, 1, 1, 1). In time step *t*_0_, the input of the neuron *N*1 is 1. The local informational context of this unit indicates that, in the five previous iterations, it has received the sequence (0, 1, 0, 1, 0). Then, when in *t*_0_ the input is processed, *N*1 recognizes *F* — i.e., the new local informational context contains the pattern (1, 0, 1, 0, 1). Therefore, in the following five time steps (*t*_1_ − *t*_5_), this neuron emits the serial binary pattern (1, 0, 1, 0, 1) with probability *p_r_*. Then, assuming that *N*1 emits *F*, the input of *N*2 in time step *t*_1_ is 1. In this case, if we assume that the local informational context of *N*2 in this time step is the sequence (0, 0, 1, 0, 0), the neuron does not recognize any fingerprint and emits the spontaneous activity with probability *p_e_*.

Using the same approach as Tristán et al. (2004), we have built networks of neurons that generate time-discrete binary signals. In these signals, 1 indicates the generation of an action potential in the corresponding time step; while 0 denotes the absence of spikes (Figure 1). Each neuron within the network is connected to other neurons according to different network topologies (see Section 2.2). These connections define the input channels of the neuron. Synapses are simple transmission channels that transfer the presynaptic output to the post-synaptic unit without any transducing mechanism. Each unit has an additional channel to introduce external stimuli in the network. This channel behaves like the synaptic channels and, therefore, all the external inputs have the same strength. The external stimulus is repeatedly delivered through the corresponding channel without silent periods between each presentation until the end of the stimulation period.

Neurons communicate by exchange of serial binary patterns. The information processing in each individual unit is oriented to determine the sequence of bits to be emitted in the following time steps in the output channels. The response pattern is calculated as a function of the detection of specific incoming stimuli. Each individual neuron has the ability to recognize a predefined set of serial binary patterns. These patterns constitute the set of neural fingerprints recognized by the neuron. In response to a recognition, the cell emits a specific output pattern in a probabilistic way. For that, neurons use *local informational contexts* (Latorre et al., 2001), i.e., local transient memories to keep track of the previous inputs. The size of each local informational context is equal to the length of the neural fingerprints considered in the corresponding simulation. These transient memories allow the implementation of a history-dependent information processing using the following rules (Figure 1):

1. Each neuron checks the recognition of a fingerprint using the corresponding local informational context in every time step, i.e., it checks whether the local informational context contains one of its known binary patterns. The external channel is checked first. In this way, the external stimulus has priority over the rest of synaptic inputs. Afterwards, the recurrent connections between neurons are checked randomly.
2. The first recognition of an input fingerprint triggers the emission of this binary pattern with probability *p_r_* (neuron *N*1 in Figure 1). With probability 1 − *p_r_*, the neuron continues processing as if no recognition happens. The probability *p_r_* varies in the different experiments. Once a neuron starts emitting an output pattern, this cannot be overridden. This implies that if the recognized fingerprint has *n* bits, no recognition takes place within the following *n* time steps.
3. If no fingerprint is recognized in a time step, the neuron emits a predefined serial binary pattern with probability *p_e_* (neuron *N*2 in Figure 1), which also varies in the different experiments (*p_e_* ≪ *p_r_*). This pattern corresponds to the *spontaneous activity* of the neuron. Note that in this situation, the neuron keeps silent with probability 1 − *p_e_*.
4. After emission, neurons have a refractory period of 10 time units during which neither emission nor recognition are made.

Probabilities *p_r_* and *p_e_* are subcellular parameters that control, accordingly, the permeability of the neuron to external stimuli and its level of spontaneous activity.

### 2.2. Network model

Many biological neural networks present structural characteristics that coincide with a small-world topology (White et al., 1986; Scannell et al., 1995; Sporns et al., 2000; Bassett and Bullmore, 2006). These networks fall between regular and random networks. In general, small-world graphs are modeled by two parameters, the connectivity and the randomness of the links (Watts and Strogatz, 1998). The main properties of the small-world networks are that they can be highly clustered like regular networks and, at the same time, have small path lengths like random ones (Watts and Strogatz, 1998; Albert and Barabási, 2002).

In this work, we build small-world networks of 2500 neurons where each unit is connected to eight other neurons. To build these networks, we start with a regular two-dimensional (50 × 50) grid with periodic boundary conditions where each unit is connected to its eight nearest neighbors. Then, each connection with a neighbor is broken with probability *p* to connect the neuron with another neuron chosen randomly (Watts and Strogatz, 1998; Lago-Fernández et al., 2000). The value of the rewiring probability *p* controls the regularity degree in the network, being the limits of regularity and randomness *p* = 0 and *p* = 1, respectively. The small-world topology lies in the intermediate region 0 < *p* < 1. As we are interested in the effect of the topological substrate on the self-organizing properties of the network, we simulate networks with different regularity degrees. In particular, networks with *p* = 0 (regular networks), *p* = 0.1, *p* = 0.25, and *p* = 1 (random networks). Hereafter, we accordingly identify these topologies with the labels “Regular,” “SW(10),” “SW(25),” and “Random.”

## 3. Results

### 3.1. Spontaneous intrinsic activity

**Figure 2:**
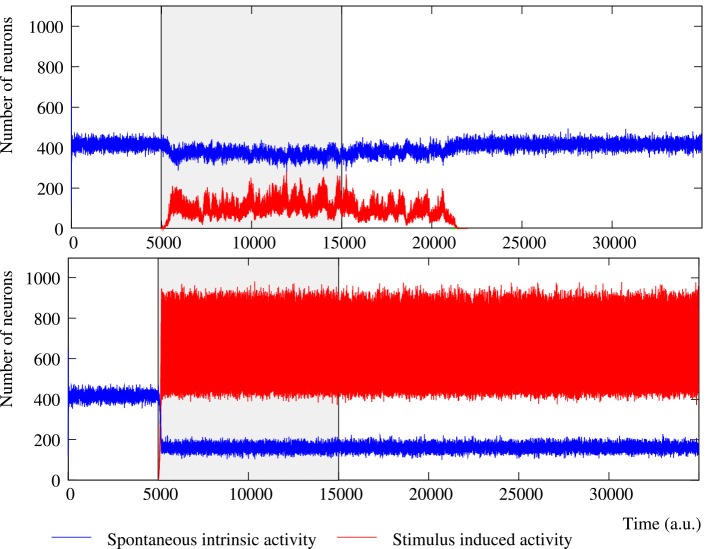
**Evolution of the collective dynamics of two different networks first without stimuli, then during the stimulation (grayed area) of a randomly chosen neuron with the stimulus (1, 0, 1, 0, 1), and finally without any stimulation again**. Results are the same with other 5-bit external stimuli. Blue traces correspond to the spontaneous intrinsic activity. Red traces show the evolution of the number of neurons that follow the external stimulus (i.e., the stimulus induced activity). The figure illustrates some of the different behaviors that the network can exhibit depending on the individual neuron parameters and the network topology (see the text for a detailed description). **Top:** Example of short-term memory network in which the global activity is nearly constant. This network consists of 2500 units with *p_e_* = 0.05 and *p_r_* = 0.35 connected with a SW(25) topology. **Bottom:** Example of long-term memory network in which the external stimulus significantly increases the level of activity in the network. The network consists of 2500 units with *p_e_* = 0.05 and *p_r_* = 0.80 connected with a SW(25) topology.

The network displays intrinsic dynamics, i.e., neural dynamics that do not directly correlate to the dynamics of an external stimulus, related to the emission of the spontaneous activity. In the absence of stimuli, the spontaneous intrinsic activity within the network evolves to a stationary state (e.g., see blue traces in the time series plotted in Figure 2 before the arrival of the external stimulus at time step 5000). For simplicity, in the simulations presented in the following sections, neurons do not recognize the spontaneous activity as a neural fingerprint. In this situation, the level of spontaneous intrinsic activity in the network only depends on the value of *p_e_* regardless the network topology (Table 1). As expected, the larger the emission probability of the spontaneous pattern, the higher the level of spontaneous intrinsic activity in the network. In simulations where the spontaneous pattern is recognized as a neural fingerprint, the network also reaches a mean steady level of spontaneous activity, but this depends on the corresponding value of *p_r_* too.

**Table 1:** **Mean number of neurons that emit the spontaneous activity (5 bits) per time step in autonomous networks of 2500 units with a given value of *p_e_* as a function of the network topology**.

***p_e_***	**Regular**	**SW(10)**	**SW(25)**	**Random**
0.05	416.64 ± 0.15	416.59 ± 0.23	416.52 ± 0.42	416.56 ± 0.24
0.10	576.96 ± 0.17	576.76 ± 0.14	576.95 ± 0.29	577.04 ± 0.38
0.15	661.72 ± 0.21	661.84 ± 0.29	661.68 ± 0.22	661.75 ± 0.22
0.50	833.34 ± 0.03	833.30 ± 0.06	833.33 ± 0.08	833.34 ± 0.06
0.80	869.57 ± 0.01	869.56 ± 0.02	869.55 ± 0.02	869.56 ± 0.03
1.00	882.42 ± 0.00	882.42 ± 0.00	882.42 ± 0.00	882.42 ± 0.00

*Mean data are calculated considering 20 simulations with different random seed, spontaneous binary pattern and connectivity. For the same value of *p_e_*, the level of spontaneous intrinsic activity in the network is nearly the same for all the network topologies. Note how precise are these values. If the spontaneous pattern has a different number of bits (from 4 to 11), results are equivalent*.

### 3.2. Detection of external stimuli

Neurons communicate by exchange of serial binary patterns. The ability of the neural network to process information based on the emission and recognition of neural fingerprints is related to the detection and propagation of specific serial patterns received through the input channels. In this section, we study the self-organizing properties of networks that receive a single stimulus, analyzing how the intraunit parameters and the network topology tune the collective neural dynamics. By default, a neuron emits a predefined spontaneous pattern. This output pattern only changes when the neuron recognizes an incoming fingerprint. In order to analyze the reverberating patterns sustained by the network and characterize the network collective dynamics, we compute during a period of time the overall number of neurons that recognize and emit a given neural fingerprint per time unit. This measure provides an estimation over time of the level of activity in the network related to each neural fingerprint. Results reported here correspond to simulations in which the network initially evolves freely and, then, the pattern (1, 0, 1, 0, 1) is introduced as external input in a randomly chosen neuron. In our first analysis, this neural fingerprint is the only pattern that neurons are able to recognized.

The simulations point out that the ability of the network to sustain the fingerprints detected in the incoming input is related to a competition between the spontaneous intrinsic dynamics and the dynamics evoked by the arrival of the external stimulus. This competition depends on a trade-off between the degree of spontaneous activity in the network and the permeability of each individual neuron to stimuli. These properties are driven, accordingly, by probabilities *p_e_* and *p_r_*. In the case of external stimuli of 5 bits, when *p_r_* is lower than 0.30 or *p_e_* is too high (see Table 2), the spontaneous intrinsic dynamics wins the competition and only the nearby neurons to the stimulated unit detect the stimulus. Thus, no significant changes are observed in the network collective activity. Table 2 compares the maximum value of *p_e_* that allows networks with different *p_r_* values to process 5-bit stimulus arriving to a single neuron. If the value of *p_e_* is above this threshold, the external stimulus does not spread through the network. The higher the value of *p_r_*, the larger the corresponding threshold of *p_e_*. This relation points out that the competition between the spontaneous intrinsic dynamics and the stimulus induced dynamics is the basis of the fingerprint-based encoding. However, the trade-off between probabilities *p_r_* and *p_e_* varies with the network topology. For a given value of *p_r_*, we observe that the corresponding threshold of *p_e_* grows with the network randomness (Table 2). This result indicates that random connections facilitate the detection and propagation of stimuli in the sense that their presence increases the “effort” required by the spontaneous intrinsic dynamics to win the competition with the stimulus induced dynamics.

**Table 2:** **Trade-off between the values of *p_e_* and *p_r_* that allows networks of 2500 units to detect 5-bit stimulus introduced into one neuron**.

***p_r_***	**Regular**	**SW(10)**	**SW(25)**	**Random**
0.30	0.01	0.03	0.04	0.04
0.50	0.07	0.09	0.10	0.11
0.80	0.15	0.18	0.20	0.21
1.00	0.20	0.25	0.26	0.28

*This value is the same regardless the predefined sequence of 5 bits used as external stimulus. Above this threshold, the network does not detect external stimuli. In the same way, when *p_r_* < 0.30, it does not detect the stimulus independently of the *p_e_* value*.

The most interesting cases from the information processing perspective are those where external stimuli propagate through the network. In these networks, a fast transition of the collective activity occurs when the stimulation begins and new collective dynamics emerges as a function of the incoming stimulus (Figure 2 at time step 5000). If we analyze the global level of activity in the network in these simulations, we observe that the preferred binary pattern reverberating in the network can follow the external stimulus. When the external stimulus arrives, the number of neurons that recognize and emit the corresponding neural fingerprint—i.e., the neurons that follow the stimulus—grows, while the spontaneous intrinsic activity drops. During the stimulation period (grayed area in Figure 2), the network evolves to a stationary state in which the stimulus induced activity fluctuates around a steady level. This steady level of activity is characteristic of the network (by network we mean the combination of network topology and intraunit parameters) and does not depend on the duration of the stimulation period. Therefore, it can be used to quantitatively characterize how external stimuli are detected and propagate through the network. As an example, Table 3 shows the corresponding characteristic value for different networks of neurons with a fixed value of *p_e_* receiving a single 5-bit stimulus. As expected, the higher the permeability of each individual unit to external stimuli (given by *p_r_*), the greater the number of neurons that follow the incoming stimulus. For the same network topology, the activity evoked by this stimulus can even be increased more than 100% by simply modifying the value of *p_r_*. For instance, in the regular network case, the mean number of neurons following the stimulus in simulations with *p_r_* = 0.5 and *p_r_* = 0.8 is around 275 (≈ 11% of the neurons in the network) and 532 (≈ 21% of the neurons), respectively. As we have already shown above, unlike the spontaneous intrinsic activity, the activity evoked by stimuli also depends on the network topology. The higher the number of random connections, the larger the detected fingerprint emission level —i.e., network randomness has a similar effect to increasing the permeability of individual neurons to external stimuli (Table 3). This results in an enhancement of the ability of the network to sustain incoming stimuli. The increased level of stimulus induced activity with the network topology is more significant for low values of *p_r_*. As probability *p_r_* grows, the level of stimulus induced activity tends to be the same for all the network topologies. For example, the difference between regular and random networks is ≈ 21% with *p_r_* = 0.5, ≈ 4% with *p_r_* = 0.8 and ≈ 2% with *p_r_* = 1.

**Table 3:** **Mean number of neurons that follow the external stimulus per time unit in networks of 2500 units with *p_e_* = 0.05 where a single 5-bit stimulus is introduced in a randomly chosen neuron**.

***p_r_***	**Regular**	**SW(10)**	**SW(25)**	**Random**
0.50	275.37 ± 1.37	318.54 ± 1.29	334.93 ± 0.96	350.62 ± 0.64
0.80	531.94 ± 0.50	540.17 ± 0.92	545.52 ± 0.57	553.52 ± 0.23
1.00	622.73 ± 0.33	628.85 ± 0.46	634.07 ± 0.56	638.02 ± 0.57

*This value characterizes the level of stimulus induced activity in the network. Fixing *p_e_* at another value, results are equivalent. Keep in mind that in the networks depicted in the table, the minimum value of *p_r_* that allows the network to detect external stimuli is 0.30 (Table 2). Mean data are calculated using 20 simulations with different random seed, 5-bit stimulus introduced in the network and connectivity*.

**Figure 3:**
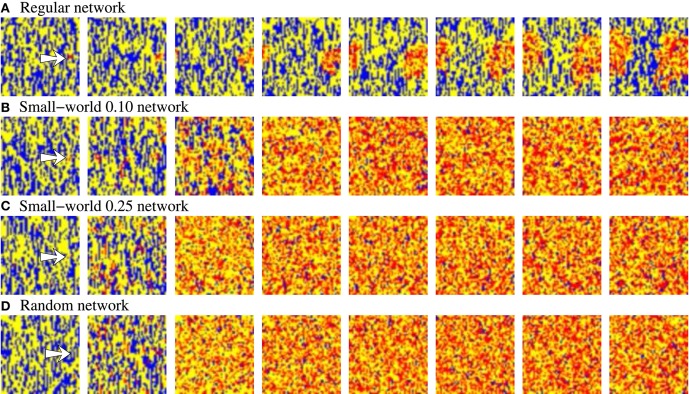
**Snapshots of four representative activity movies illustrating the external stimulus propagation**. The four networks have an equivalent behavior in terms of level of intrinsic spontaneous activity and level of activity evoked by external stimulus. To better appreciate the difference between the different topologies, we show networks with a high level of stimulus induced activity. Sequences develop in time from left to right with a time interval between frames of 20 a.u. Each point in the 50 × 50 square represents with a color code the evolution in time of a neuron within the network. Yellow corresponds to silent neurons (i.e., neurons not emitting a specific binary pattern). Blue corresponds to the spontaneous intrinsic activity. Red corresponds to the emission of the pattern associated to the external stimulus. The arrow in the first frames indicates the approximate location of the stimulated neuron. Note that the spontaneous intrinsic activity decreases as a consequence of the propagation of the stimulus until the preferred pattern reverberating in the network follows the external stimulus (in the regular network, this situation is not observed in the snapshots due to the slower propagation velocity).

In addition to the level of activity induced by the external stimulus, another relevant feature characterizing how information spreads through the network is the stimulus propagation velocity. This can be estimated using the time the network needs to reach its characteristic steady level of stimulus induced activity from the beginning of the stimulation. Beyond the expected effect of *p_r_*, if we analyze the propagation velocity in networks with an equivalent behavior in terms of level of spontaneous intrinsic activity and stimulus evoked activity (e.g., see Table 4), we observe that the higher the network randomness, the faster the stimulus propagation through the network. The difference on the propagation velocity as a function of the network topology can be explained analyzing how stimuli travel through the network. To illustrate the external stimulus propagation, we have generated square-shaped movies representing the evolving network dynamics. In these activity movies, each point in the 50 × 50 frame represents with a color code the activity of a neuron, i.e., the binary pattern that emits in the corresponding time step. Figure 3 shows snapshots of four representative activity movies illustrating how an external stimulus propagates through networks with different topologies. In regular networks (panel A), the stimulus spreads as wave fronts centered in the stimulated unit. Then, to reach the furthest regions, it needs to travel through the whole network. Conversely, in less regular topologies (panels B–D), the stimulus is distributed through the whole network almost from the beginning of the stimulation. This produces several coexisting propagating fronts of information. The presence of a single wave front in regular networks translates into a slower propagation velocity.

**Table 4:** **Mean external stimulus propagation velocity (*neurons/time*) as a function of the network topology in networks of 2500 units with *p_e_* = 0.05 and *p_r_* = 0.80 where a single 5-bit stimulus is introduced in a randomly chosen neuron**.

**Topology**	**Propagation velocity**
Regular	3.82 ± 0.44
SW(10)	13.15 ± 2.11
SW(25)	16.88 ± 1.30
Random	17.80 ± 1.13

*Regardless the network topology, these networks achieve nearly the same level of spontaneous intrinsic activity (Table 1) and stimulus induced activity (Table 3). With other parameters, results are equivalent but varying the mean propagation velocity. Mean data are calculated considering 100 simulations with different random seed and 5-bit stimulus introduced in the network*.

**Figure 4:**
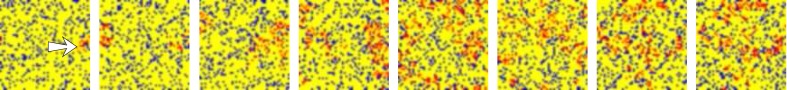
**Snapshots of an activity movie belonging to the network depicted in top panel of Figure 2**. The figure illustrates the spatio-temporal patterns generated by the network in response to stimulus. Sequences develop in time from left to right with a time interval between frames of 50 a.u. Color code is the same used in Figure 3. The arrow in the first frame indicates the approximate location of the stimulated unit.

The activity movies representing the evolving network dynamics point out that not only the level of activity evoked by stimulation characterizes the fingerprint-based encoding. Localized patterns of activity traveling through the network with different spatial organization can be formed due to the external stimulus propagation. These spatio-temporal patterns generated in response to stimuli can remain bounded in a region, can propagate with a fix spatial structure or as transient fronts of information, or can lack a well-defined spatial structure. Figure 4 shows a representative example of these spatio-temporal patterns in a network with a lower synchrony degree than in networks depicted in Figure 3. The generation of transient patterns of activity is crucial for the detection and encoding of external stimuli because, as we discuss in Section 3.5, the coexistence of several stimuli within the network is related to the different clusterization and coherence of these stimuli evoked spatio-temporal patterns.

Results presented in this section correspond to stimuli of 5 bits introduced in a randomly chosen neuron. In simulations where external stimuli have a different length (from 4 to 11 bits) and/or they are injected in a greater number of neurons (from 2 to 30), results are equivalent to the ones discussed here, but taking into account the following. On one hand, increasing stimulus lengths helps the spontaneous intrinsic activity to win the competition because longer stimuli require more time steps to be detected and, therefore, the probability to emit the spontaneous binary pattern increases. And, on the other hand, a greater amount of stimulated neurons helps the detection process since the level of activity related to the external stimulus grows with the number of stimulation sources.

### 3.3. Network memory

In the previous section, we have studied the ability of the neural network to sustain external stimuli while they are active. However, an interesting feature of the network is its memory ability, i.e., the ability to sustain a detected pattern in the collective fingerprint-based dynamics beyond the time period of stimulation. In order to study this property, we have carried out simulations where the external stimulus is introduced in the network just for a while. In these simulations, we study how the stimulus induced activity survives in the network after the end of the stimulation. Taking into account the results described in Section 3.2, we focus on networks able to detect incoming stimuli. Here, we analyze the same simulations as in the previous section, but when at time step 15,000 the stimulation ends—i.e., the network initially evolves freely, then, at time step 5000, the stimulus (1, 0, 1, 0, 1) is introduced into a randomly chosen neuron during 10,000 steps, and, finally, no stimulation is present again (Figure 2). Equivalent results to those presented here are obtained in simulations with external stimuli from 4 to 11 bits injected in a greater number of neurons (from 2 to 30).

The level of activity analysis indicates that the network may sustain external stimuli transiently—i.e., the stimulus induced activity reverberates just for a while and then the corresponding binary pattern disappears from the network (short-term memory or working memory)—or persistently—i.e., after the stimulation, the activity evoked by the stimulus coexists with the spontaneous intrinsic activity in a permanent way (long-term memory). Top and bottom panels of Figure 2 illustrate, correspondingly, two examples of short-term and long-term memory. In short-term memory networks, the propagation of the external stimulus is a transient effect directly linked to stimulation. Thus, when the stimulation ends and no external input supports the generation of the spatio-temporal patterns encoding the stimulus, the corresponding activity stepwise disappears from the network and the spontaneous intrinsic activity prevails (i.e., the network “forgets” the stimulus). Stimuli usually reverberate in short-term memory networks with a regular topology for longer periods than in random networks, in which stimuli can even disappear almost instantaneously when the stimulation is over. However, no general conclusions can be drawn because the stimulus reverberation period significantly varies even in the same network receiving the same stimulus (cf. transition periods between external stimuli in **Figure 6**). In long-term memory networks, the stimulation produces persistent changes in the network collective dynamics. This leads the network to a new stable state where the reverberation of the patterns associated with the external stimulus is not sustained by stimulation, but it is a network effect. In this way, external stimuli can win the competition and prevail over the spontaneous intrinsic activity (e.g., see final snapshots of Figure 3). In these cases, the spontaneous activity does not completely disappear from the network because of the probability of emitting this pattern when no fingerprint is recognized in a time step (*p_e_*).

**Figure 5:**
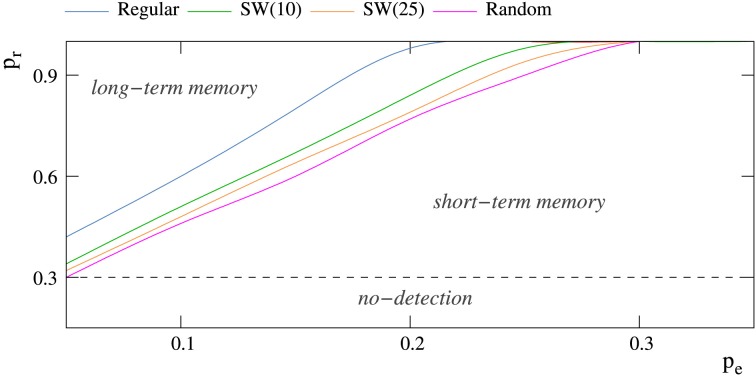
**Phase diagram showing the regions at which short-term and long-term memory phases appear for networks of 2500 neurons receiving a single 5-bit stimulus**. The minimum probability *p_r_* that allows these networks to detect external stimuli is 0.30 (Table 2). Each color trace corresponds to networks with a different topology.

The factors that determine if the neural network behaves as a short-term or a long-term memory are again related to the competition among the spontaneous intrinsic and the stimulus induced activity. As we have previously discussed in Section 3.2, the mode of competition varies as a function of the relation among the intraunit parameters *p_e_* and *p_r_* and the network topology. To illustrate the relationship between these factors, a phase diagram locating the different behaviors in the space of intraunit parameters for networks with different topologies is shown in Figure 5. There is a threshold value of *p_r_* for each value of *p_e_* that determines if the network behaves as a transient (below the threshold) or a persistent memory (above the threshold). Independently of the network topology, as the level of spontaneous intrinsic activity grows, a higher permeability to external stimuli is required to become a long-term memory. Regarding the network topology, random connections contribute to long-term memory mechanisms, unlike regular connections that help short-term memory mechanisms. In this manner, for example, when *p_e_* = 0.05, random enough (*p* > 0.80) small-world networks of 2500 units never behave as a short-term memory.

### 3.4. Total activity in the network

The network can display two different modes of operation according to the evolution of the total level of activity in the network, i.e., the overall number of active neurons whatever the binary pattern they are emitting. In Section 3.1, we show that the spontaneous intrinsic activity in autonomous networks reaches a steady level. Similarly, the arrival of a stimulus makes the level of activity related to this external input also reaches a steady level. In these cases, a transition to a lower level of spontaneous activity is produced due to the spreading of the stimulus (see Section 3.2 for details). This lower level is also kept constant during the stimulation period. Nevertheless, the total level of activity in the network can be kept nearly constant over time, but also it can be increased due to stimulation (cf. Figure 2, top and bottom panel). Networks with a constant level of total activity seem to have a maximum processing capacity in terms of the maximum number of neurons that can be simultaneously active. In the presence of stimuli, active neurons are distributed between the different binary patterns present in the network. The arrival of a stimulus produces a proportional decrease in the spontaneous intrinsic activity as the increase in the stimulus induced activity, and vice versa when the stimulation ends. Only short-term memory networks display this mode of operation. In contrast, when the stimulus increases the level of activity in the network, the number of neurons that follow the stimulus grows faster than the decreasing spontaneous activity. In these cases, the network can sustain the stimulus permanently (as in the example of Figure 2) or transiently. In this last situation, once the external stimulus disappears from the network, this recovers the steady level of spontaneous intrinsic activity.

### 3.5. Encoding of multiple simultaneous stimuli

A major point of interest in this study is the network response to multiple stimuli, i.e., the encoding and coexistence of several neural fingerprints in the network. In this section, we discuss the emerging collective dynamics of networks receiving external stimuli in series (Section 3.5.1) and in parallel (Section 3.5.2). The arrival of multiple stimuli simultaneously or close in time induces the generation of different coexisting patterns of activity within the network. In this situation, a competition between the spatio-temporal patterns encoding the different stimuli arises. In simulations with in-series stimulation, we study the competition between reverberating patterns encoding a previously received stimulus and patterns supported by an active stimulus. This allows us to assess the ability of the network to retain information regarding recent past stimuli (i.e., its transient memory capability) when a new stimulus arrives. Meanwhile, in-parallel stimulation allows studying the ability of the network to sustain several simultaneous stimuli.

To address the effect of network topology on the spatio-temporal activity of the network, we compare the self-organizing properties of “equivalent networks” in terms of spontaneous intrinsic activity (Section 3.1), stimuli induced activity (Section 3.2), type of memory (Section 3.3), and total activity in the network (Section 3.4). This allows us to compare networks with different topologies under the same conditions. We discuss here two particular cases of equivalent networks:

- Short-term memory networks with a low level of activity. These networks generate propagating well-defined spatio-temporal patterns to encode incoming stimuli (e.g., see Figure 4). Then, one could intuitively expect a slight competition between coexisting spatio-temporal patterns, which a priori must contribute to a better encoding of multiple simultaneous stimuli.
- Long-term memory networks with a high level of activity. When a long-term memory network detects an external stimulus, the corresponding spatio-temporal activity is sustained by the network intrinsic dynamics. Furthermore, a high level of activity induces almost total synchronization over the whole network (e.g., see Figure 3). Therefore, under these conditions, a strong competition among coexisting patterns of activity must a priori arise in the network.

In the following sections, we discuss equivalent networks of 2500 neurons with the intraunit parameters shown in Table 5 and receiving 5-bit stimuli. For other equivalent networks receiving stimuli of a different length (from 4 to 11 bits), results are equivalent.

**Table 5:** **Values of *p_e_* and *p_r_* (respectively, first and second value of each pair) used in the simulations presented in Sections 3.5.1, 3.5.2**.

**Topology**	**Short-term memory**	**Long-term memory**
Regular	0.05 - 0.42	0.05 - 0.80
SW(10)	0.05 - 0.34	0.05 - 0.80
SW(25)	0.05 - 0.32	0.05 - 0.80
Random	0.05 - 0.31	0.05 - 0.80

*When a network of 2500 units with the parameters of the first column receives a stimulus of 5 bits in a single neuron, its mean level of spontaneous intrinsic activity and level of stimulus induced activity are ≈410 and ≈120 neurons per time unit, respectively. Therefore, they are equivalent short-term memory networks with a low level of activity. Similarly, networks of 2500 units with the parameters of the second column where stimuli are injected in clusters of 2 × 5 neurons are equivalent long-term memory networks with a high level of activity (their level of intrinsic spontaneous activity and their level of stimulus induced activity are ≈410 and ≈690 neurons per time unit)*.

#### 3.5.1. In-series stimulation

In simulations where the stimuli are in series introduced in the network, we alternate stimulation episodes in which two different external stimuli are injected in the same group of neurons. In the simulations presented here, initially, the network evolves freely. At time step 5000, the stimulation begins. In each stimulation episode, a single stimulus arrives to the network during 10,000 time units. Then, there is an inter-stimulation interval of 5000 time steps where no stimulus is introduced. This sequence is repeated eight times alternating two different stimuli (A and B), which are the two binary patterns recognize by the neurons in the network. Finally, at time step 120,000 no stimulus is introduced any longer. Results are independent of the stimulation patterns and of the location of the neurons that receive the external stimuli.

**Figure 6:**
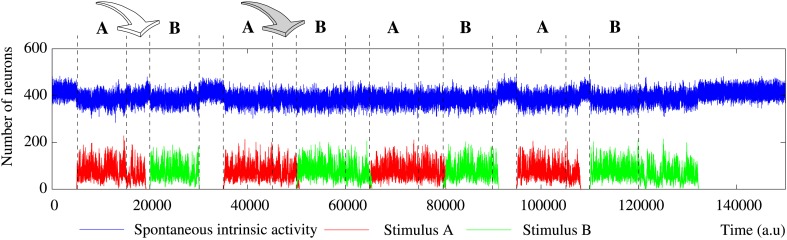
**Evolution of the activity of a SW(10) short-term memory network that receives two stimuli in series**. The network consists of 2500 neurons with the intraunit parameters shown in Table 5. Stimulus A is (1, 0, 1, 0, 1) and stimulus B (1, 1, 0, 1, 1). Labels on top indicate the stimulus injected in each stimulation episode. The figure plots the evolution of the spontaneous intrinsic activity (blue trace) and the emission level of the fingerprints associated to stimulus A (red trace) and B (green trace). In short-term memory networks, small differences resulting of the network topology exist on the network collective dynamics. If the stimulus detected in a prior episode survives in the network when a new stimulation episode starts, the new stimulus almost instantaneously wins the competition (winner-take-all competition) and the previous stimulus completely disappears from the network.

**Figure 7:**
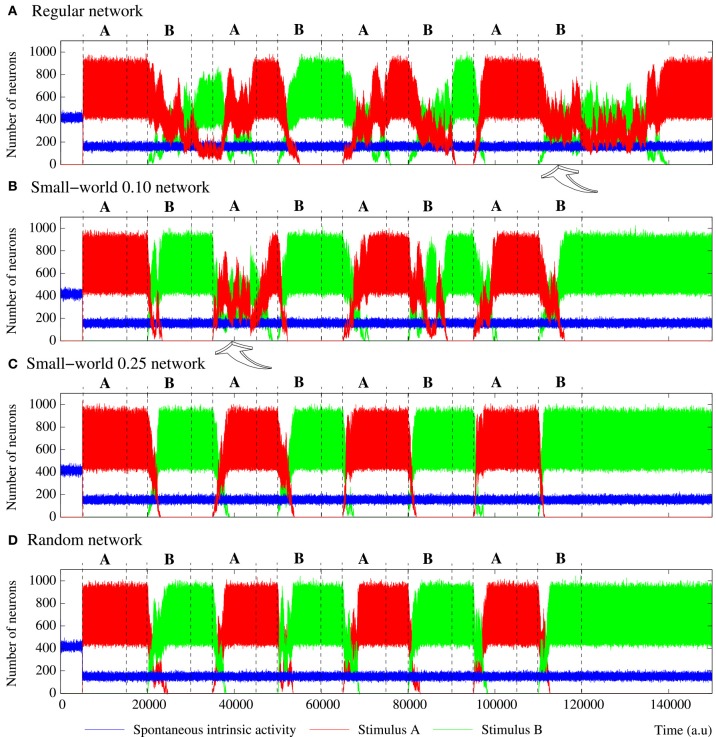
**Equivalent figure to Figure 6, but for equivalent long-term memory networks with different topologies**. These networks consist of 2500 units with the parameters shown in Table 5. Again, stimulus A is (1, 0, 1, 0, 1) and stimulus B (1, 1, 0, 1, 1). During the stimulation periods, a competition is established between stimuli A and B. In networks with a high degree of regular connections **(A,B)**, this can be a winner-take-all or a winnerless competition. In the more random networks **(C,D)**, it is always a winner-take-all competition where the activity supported by the corresponding active stimulus prevails.

Equivalent results to the ones described in the previous sections are also produced when the network receives two stimuli in series (see Figures 6, 7). These results can be summarized in the following points:

- Initially, without any stimulus, the activity of the network evolves to a stationary state where only spontaneous intrinsic activity is present in the network regardless its topology.
- The total activity in the network can be kept constant or be increased due to stimulation depending on the intraunit parameters and the network topology.
- Stimulation evokes collective dynamics in which the spontaneous intrinsic activity competes with the activity induced by external stimuli. Due to this competition, the level of spontaneous intrinsic activity drops.
- When the stimulation is over, reverberating patterns encoding external stimuli can survive within the network transiently or persistently.

However, in-series stimulation induces new collective dynamics regarding the competition between stimuli A and B. In the case of equivalent short-term memory networks, no significant differences are found in their collective dynamics. As example of activity in these networks, Figure 6 depicts the activity of a representative short-term memory network with SW(10) topology that receives two stimuli in series. In many occasions, mainly in the most random networks, the activity evoked by a stimulus disappears from the network during the subsequent inter-stimulation interval. Then, when a new stimulation episode starts, the network has recovered a stable state with only spontaneous intrinsic activity and no competition between external stimuli occurs. This situation can be observed in different transition periods in Figure 6, for instance, in the pointed out by the white arrow. In other stimulation episodes, mainly in the more regular networks—where external stimuli reverberate for longer periods (see Section 3.3)—a competition takes place between stimuli A and B. A priori, given the spatial organization of the patterns induced by stimuli in short-term memory networks with a low level of activity (e.g., see Figure 4), we could expect that reverberating patterns encoding the stimuli transiently coexist within the network. Nevertheless, a fast winner-take-all competition always occurs between both stimuli, and the last received stimulus quickly prevails over the other (e.g., transition period pointed out by the gray arrow in Figure 6). Once one of the external stimuli wins the competition, the number of neurons that recognize and emit the corresponding binary pattern fluctuates around a steady level. This steady level is nearly the same in all the stimulation episodes since neurons have the same permeability to stimulus A and B. Finally, when the stimulation ends, the last injected stimulus can reverberate in the network in the same way as in the single stimulus case.

More interesting results from the encoding point of view appear in the simulations of long-term memory networks receiving external stimuli in series (Figure 7). In these networks, once an external stimulus is detected, it persistently survives in the network. Therefore, given the stimulation protocol used in our simulations, a competition between the two incoming stimuli always happens when a new stimulation episode begins. Due to this competition, the time needed to reach a steady level in the number of neurons that follow each incoming stimulus is very variable in the different transition periods. Note also the difference in the transition of the collective activity as compared with the first stimulation episode where no competition between external stimuli is established (*p_r_* ≫ *p_e_*). As we demonstrate in Section 3.2, the presence of random connections increases the level of activity induced by an external stimulus and the corresponding propagation velocity. When two stimuli are in series introduced into a random enough long-term memory network (e.g., a SW(25) network), this results in a relatively fast (as compared with networks with more regular topologies) winner-take-all competition where the activity supported by the active stimulus in each stimulation episode prevails. Thus, in networks with prevailing random connections, the last received stimulus always wins the competition and, therefore, the preferred pattern reverberating in the network starts following this stimulus (panels C, D of Figure 7). Conversely, the presence of regular connections benefits the competition between external stimuli (Section 3.2). Due to this phenomenon, it can be visually appreciated that when a winner-take-all competition among stimuli A and B takes place in more regular networks (see panels A, B of Figure 7), the last injected stimulus usually needs more time to win the competition than the required in the more random networks. Table 6 quantifies this result showing the mean time elapsed since the arrival of a new external stimulus until the binary pattern associated to the previous stimulus completely disappears from long-term memory networks with different topologies. In networks exhibiting winner-take-all competitions the arrival of an specific but minor stimulus in the network induces an alternation of activity in the whole ensemble.

**Table 6:** **In long-term memory networks, stimuli survive in the network until a new stimulus arrives**.

**Regular**	**SW(10)**	**SW(25)**	**Random**
10,477.75 ± 4500.46	5759.95 ± 4376.25	3391.66 ± 1650.48	3226.42 ± 1,167.10

*The table shows the mean time that the long-term memory networks depicted in Figure 7 need to “forget” a previously detected stimulus. This period is calculated from the beginning of a new stimulation episode to the instant where the level of activity related to the previous stimulus is equal to zero. Units are dimensionless. Mean data are calculated considering 20 simulations with different random seed, 5-bit stimuli introduced in the network, stimulated unit and connectivity. The higher the network regularity, the longer the competition period between external stimuli*.

**Figure 8:**
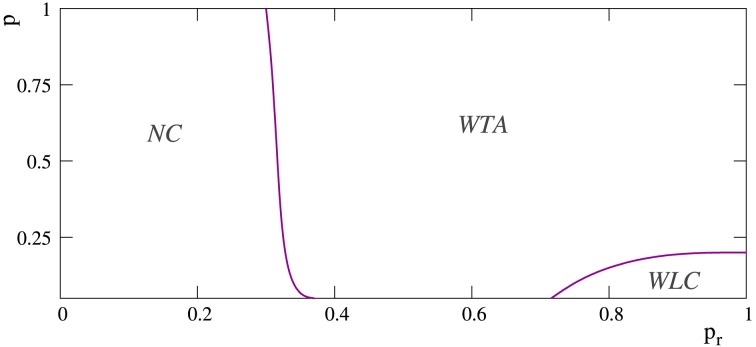
**Phase diagram depicting the regions at which the different competition regimes emerge in networks of 2500 neurons with *p_e_* = 0.05**. The x-axis corresponds to probability *p_r_* and the y-axis to the rewiring probability parameter *p*, i.e., to the network topology. Note that *p* = 0 corresponds to regular networks and *p* = 1 to random networks. Label WLC denotes regions where transient winnerless competitions between external stimuli can appear in the network. Label WTA denotes regions where the mode of competition between external stimuli is always a winner-take-all regime. The region NC corresponds to networks where no competition among external stimuli takes place.

However, in the more regular networks, the mode of competition among external stimuli is not always a winner-take-all regime. As illustrated in the stimulation episodes pointed out by the arrows in panels A, B of Figure 7, transient winnerless competitions (Rabinovich et al., 2001; Afraimovich et al., 2004) can be established in these networks. Figure 8 shows a phase diagram depicting the regions where winner-take-all and winnerless competition regimes emerge in long-term memory networks of 2500 units with *p_e_* = 0.05 as a function of the network topology as given by the rewiring probability parameter and the value of the intraunit parameter *p_r_*. During winnerless regimes, no stimulus wins the competition and, therefore, both external stimuli coexist in the network although one of them is not supported by stimulation. Due to this competition dynamics, two interesting phenomena can be observed in the more regular long-term memory networks when the stimulation is over. On one hand, the persistently sustained stimulus is not necessarily the injected in the last stimulation episode. Panel A of Figure 7 shows an example of this effect. Although stimulus B arrives in the last stimulation episode, the stimulus that survives in the network after the end of the stimulation is stimulus A. On the other hand, a sustained winnerless competition between external stimuli can emerge in the network (this is illustrated above in **Figure 13** for a network receiving nine external stimuli simultaneously). Therefore, these networks have the ability to behave as a long-term memory simultaneously encoding more than one stimulus.

**Figure 9:**
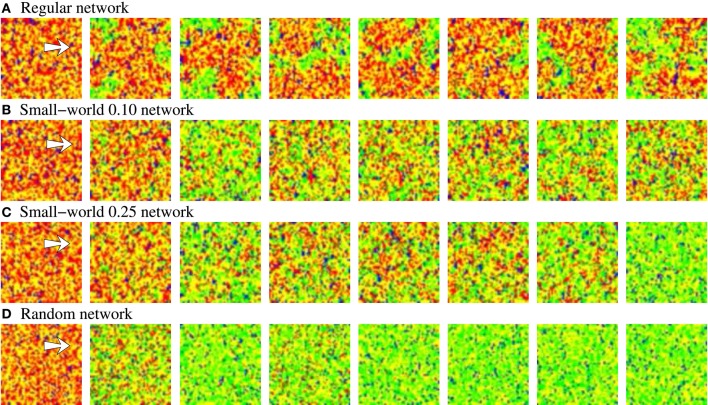
**Snapshots of activity movies belonging to the networks depicted in Figure 7**. The figure illustrates the evolving spatio-temporal patterns of activity observed in these networks as a function of the different modes of competition established between stimuli A and B when a new in-series stimulation episode starts. Sequences develop in time from left to right. To better appreciate the different evolving spatial structure of the patterns, the time interval between frames is different, but always the same in the four sequences. Neural activity is represented with a color code. Yellow corresponds to silent neurons, blue to the spontaneous intrinsic activity, red to stimulus A and green to stimulus B. The arrow in the first frame of each sequence points out the approximate location of the 2 × 5 stimulated cluster of neurons. When stimulus B arrives, it propagates through the network. Then, as the activity related to this stimulus grows, the activity related to stimulus A diminishes. When a winner-take-all competition occurs **(C,D)**, stimulus B completely replaces to stimulus A. The higher the regularity of the network, the longer the transition period between stimuli. When a winnerless competition is established between stimuli A and B, both stimuli coexist in the network **(A,B)**.

The activity movies illustrating the evolving network dynamics give additional insight about the results derived from the level of activity analysis. When a new stimulation episode starts, the new stimulus induces the coexistence of different spatio-temporal patterns with different spatial organization. Figure 9 shows the evolving patterns generated by four representative examples of equivalent long-term memory networks during an inter-stimulation interval. The four sequences start just before the arrival of stimulus B, in a situation with a high level of synchrony in the network where the prevailing pattern corresponds to stimulus A (red points). The arrival of stimulus B (second frame) produces new emerging spatio-temporal patterns, and a competition among the two external stimuli starts. As Figure 7 shows, this competition depends on the network topology. Snapshots of Figure 9 can explain the transient winnerless competition dynamics observed in the more regular networks, and the winner-take-all dynamics in the more random ones. In the more regular networks (panels A, B of Figure 9), the evolving spatio-temporal patterns have a well-defined and coherent spatial structure. The clusterization and coherence of these transient patterns produce well-delimited boundary regions between stimuli A and B, which potentiates the competition. This improves the ability of the network to sustain multiple stimuli simultaneously. In the same way, the winner-take-all competition observed in the more random networks in Figure 7 is also observed in the snapshots of Figure 9. Note that the unstructured propagation of stimulus B makes it quickly replaces stimulus A within the network, reaching the typical fix spatial organization of the pattern.

#### 3.5.2. In-parallel stimulation

In the simulations where multiple external stimuli arrive in parallel to the network, we consider a single stimulation episode. During this time period, all the external stimuli considered in the experiment are simultaneously introduced in different groups of neurons. The set of neural fingerprints recognized by the neurons varies in the different experiments, but always consists of the serial binary patterns used as external stimuli during the corresponding stimulation period. Results are independent of the stimulation patterns and of the location of the neurons that receive the external stimuli.

**Figure 10:**
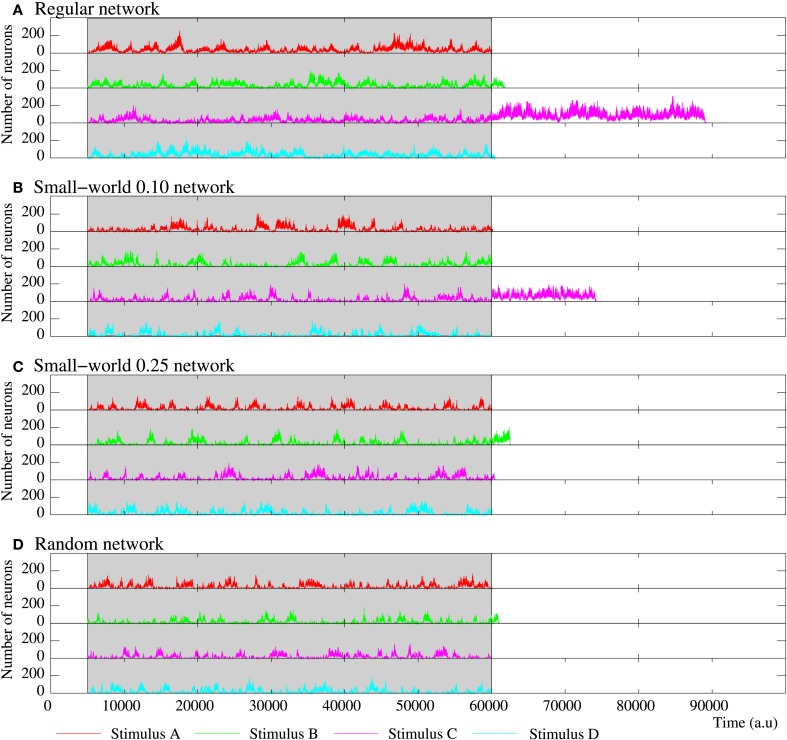
**Evolution of the activity of four equivalent short-term memory networks receiving four stimuli in parallel**. All of them consist of 2500 neurons with the parameters shown in Table 5. Stimulus A is (1, 0, 0, 0, 1), B (1, 0, 1, 0, 1), C (1, 1, 0, 1, 1), and D (1, 1, 1, 1, 1). The network collective dynamics is the same independently of the serial binary patterns used as external stimuli. The figure plots the number of neurons following each stimulus during and after the stimulation period (grayed area). Note that the spontaneous intrinsic activity is not shown. While the stimulation is present, a winnerless competition between the four stimuli arises within the network. When the stimulation ends, since they are not supported by an active stimulus, the binary patterns associated to the external stimuli disappear from the network after a reverberation period. As in all the previously discussed short-term memories, the more regular networks are able to sustain external stimuli for longer periods when the stimulation is over.

**Figure 11:**
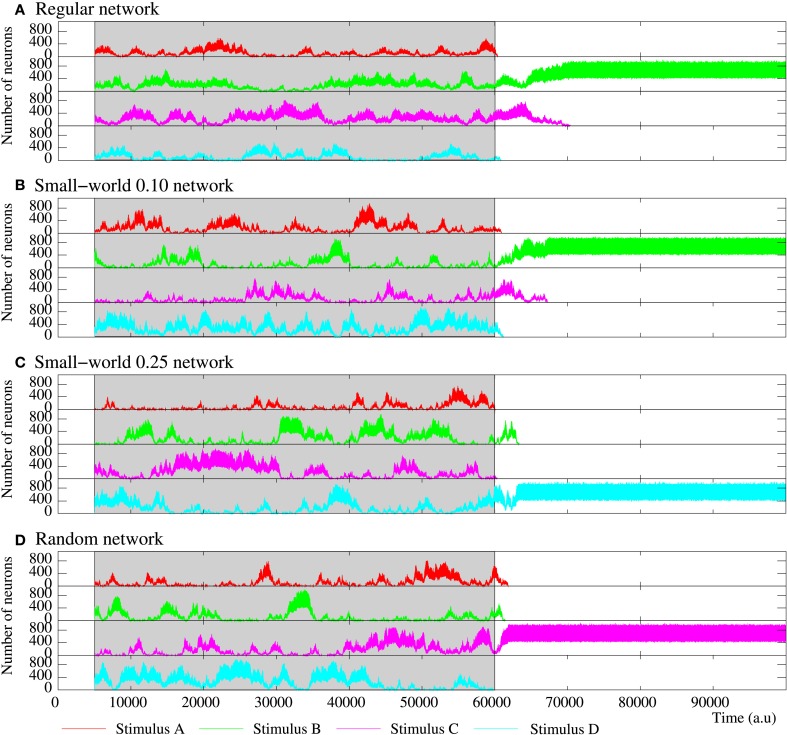
**Figure equivalent to Figure 10, but for four representative examples of equivalent long-term memory networks of 2500 neurons with the intraunit parameters shown in Table 5**. Stimulus A is (1, 0, 0, 0, 1), B (1, 0, 1, 0, 1), C (1, 1, 0, 1, 1), and D (1, 1, 1, 1, 1). During the stimulation period, a sustained winnerless competition emerges in the network. This consists of an irregular alternation of the level of activity related to each external stimulus. When the stimulation is over, at least a stimulus survives in the network.

**Figure 12:**
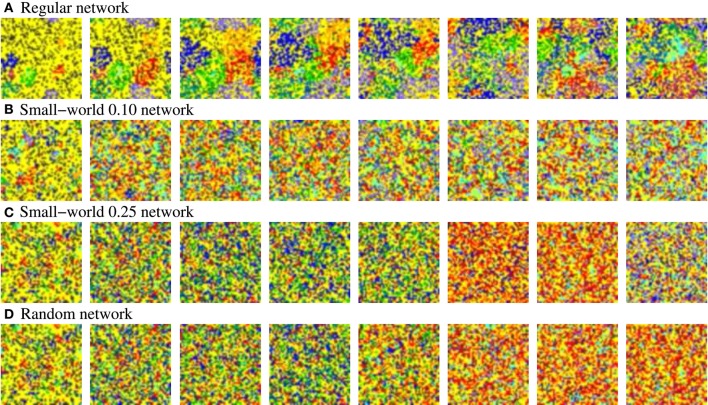
**Snapshots of four representative activity movies of equivalent long-term memory networks with different network topologies receiving nine 9-bit stimuli in parallel**. Similar patterns of activity are obtained in short-term memory networks. Sequences develop in time from left to right. To better appreciate the spatial organization of the patterns, the time interval between frames in the sequence is variable, but always the same in the four sequences. Neural activity is represented with a color code. In this case, yellow corresponds to silent neurons or neurons emitting the spontaneous binary pattern. The rest of colors correspond to a different external stimulus. Note that we do not distinguish between silent neurons and neurons emitting the spontaneous pattern to simplify the graphical representation and better appreciate the evolution of the patterns. The figure shows the characteristic spatial organization of the patterns as a function of the network topology. In the more regular networks **(A,B)**, several coherent spatio-temporal patterns coexist within the network. Conversely, in the more random networks **(C,D)**, the number of coexisting patterns in a given time step is lower.

When stimuli are applied, they start to spread through the network and, again, new collective dynamics emerge in the network (Figures 10, 11). If we focus on the stimulation period, the same as in all the simulations described so far, on one hand, the spontaneous intrinsic activity (not shown in Figures 10, 11) drops due to the competition with the activity induced by incoming stimuli and, on the other hand, the total level of activity in the network reaches a steady level. However, as now multiple simultaneously active stimuli support their generation, coexisting patterns encoding the different external stimuli propagate through the network and compete between them. The same as in the in-series stimulation case (Section 3.5.1), the competition dynamics among the coexisting patterns of activity is the basis of the encoding of multiple stimuli. While the stimulation is present, now, it always arises a winnerless competition consisting of irregular intervals where the overall activity within the network is distributed in turn between the different external stimuli. In this sense, none of the stimuli neither prevails over the others nor reaches a steady emission level during the stimulation period. Depending on the connectivity, different winnerless regimes can arise in the network. Note that when the network is in-series stimulated, winnerless competition dynamics only arises in the more regular topologies. The activity movies illustrate the different spatial organization of the coexisting patterns of activity as a function of the network topology, which allows us to understand the different winnerless regimes. Snapshots of the evolution of the network activity of four equivalent long-term memory networks with different topologies are shown in Figure 12. When the network has a high degree of regular connections, coherent spatio-temporal patterns are formed due to the propagation of stimuli to close neighbors. In these networks, there are well-defined clusters of neurons that follow each stimulus and, therefore, it is established a winnerless competition where each external stimulus keeps a nearly constant level of activity. If one stimulus disappears from the network, new spatio-temporal patterns following it appear shortly after due to the stimulation. Conversely, in networks with a high level of random connections, the presence of connections between distant neurons produces patterns of activity with a lower spatial structure and a faster propagation velocity. These two features induce a competition between the stimuli induced activity that makes a few stimuli (in the examples of Figures 10, 11, only one or two) transiently prevail over the others. This translates into a winnerless competition regime consisting of the alternation of irregular cycles where the preferred pattern in the network follows different stimuli. Note how in the snapshots of panels C, D in Figure 12, the prevailing colors in each frame change as time evolves, while in panels A, B the color distribution is homogeneous. The lower the randomness, the higher the number of coexisting spatio-temporal patterns within the network. Therefore, connections to close neighbors increase the ability of the network to sustain multiple incoming stimuli simultaneously. Table 7 corroborates this result by calculating the mean number of coexisting stimuli in equivalent long-term memory networks during an in-parallel stimulation where nine stimuli are simultaneously applied.

**Table 7:** **Mean number of stimuli encoded during the stimulation period in the networks shown in Figure 12 when they receive nine simultaneous 9-bit stimuli**.

**Regular**	**SW(10)**	**SW(25)**	**Random**
7.77 ± 0,95	6.68 ± 1,18	4.51 ± 1,19	4.29 ± 1,11

*We consider that the network encodes a stimulus when the number of neurons that follow it is greater than ten (i.e., the number of stimulated cells). Mean data are calculated considering 20 different simulations with different random seed, external stimuli introduced in the network and connectivity*.

**Figure 13:**
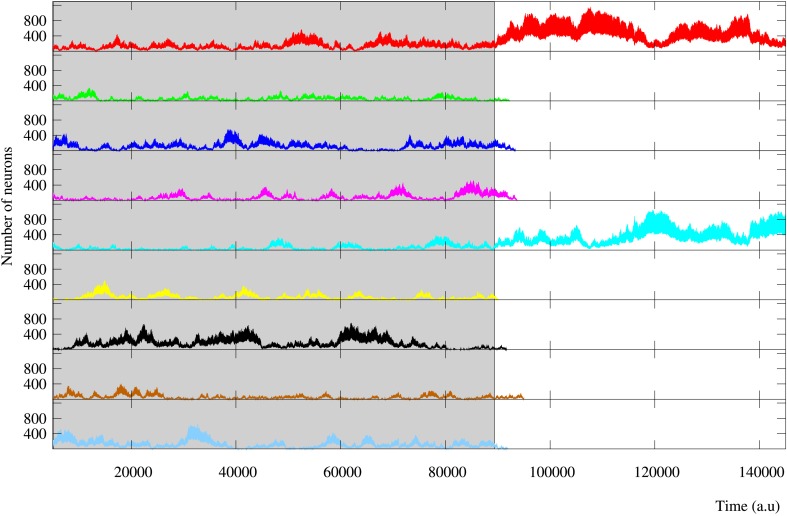
**Evolution of the activity of a regular long-term memory network where nine external stimuli are injected in parallel**. Each trace corresponds to a different external stimulus using the same color code as in Figure 12. Grayed area identifies the stimulation period. This figure illustrates how connections to close neighbors promote the competition between the activity induced by the different external stimuli. This implies that these networks are able to encode a larger number of stimuli simultaneously, even after the stimulation period when they are not supported by an active stimulus.

Finally, results observed in short-term memory networks when the stimulation is over are equivalent to those obtained during in-series stimulation. In long-term memory networks, a winner-take-all competition usually occurs and the reverberating patterns follow one of the external stimuli (Figure 11). Nevertheless, in some simulations of regular long-term memory networks a sustained winnerless competition between two or three stimuli is established in the network beyond the stimulation period. Figure 13 illustrates this encoding mechanism in a long-term memory network that receives nine different stimuli. This result emphasizes the increasing ability to encode multiple simultaneous stimuli that the presence of regular connections provides to the neural network.

## 4. Discussion

The idea that neural systems can encode information in the precise timing of spikes has attracted increasing attention over the last years. The presence of precise firing patterns in different neurons has been reported in several vertebrate and invertebrate neural circuits (Elson et al., 1999; Reinagel and Reid, 2000, 2002; Chi and Margoliash, 2001; Hunter and Milton, 2003). Experimental evidence demonstrates that some of these precise temporal structures allow the discrimination of neural signals. For instance, the bursting activity of some neurons belonging to very different animals displays intraburst neural signatures in the form of cell-specific interspike interval distributions (Szücs et al., 2003, 2005; Garcia et al., 2005; Campos et al., 2007; Zeck and Masland, 2007). Similarly, some neural systems can display specific firing patterns associated to behavioral or functional states (Klausberger et al., 2003; Somogyi and Klausberger, 2005; Kaping et al., 2011). However, although there is an increasing amount of new results on the strategies of information processing used by neural systems (VanRullen et al., 2005; Rabinovich et al., 2006; Kumar et al., 2010), the existence of characteristic features in an individual neural signal allowing a reader to discriminate its inputs has not been investigated in great detail. This kind of information processing can be a powerful strategy for neural coding. Model simulations support the hypothesis that intraburst neural signatures could be part of a multiplexed code where the neuron identity could be transmitted together with the circumstantial message (Latorre et al., 2006, 2007). Readers of these signals can take advantage of these multiple simultaneous codes and process them one by one or simultaneously to perform different tasks (Latorre et al., 2006; Baroni et al., 2010). Thus, if a neural system is able to recognize different neural fingerprints in its input signals and adjust its behavior to them, it could discriminate or contextualize their inputs as a function of general aspect of the signal like specific interspike frequencies via resonance (Izhikevich et al., 2003;), but also as a function of a specific emitter or task. This is a very desirable ability in multifunctional systems.

This work presents a simulation study showing that a simple neural network model composed of neurons that are able to emit and recognize neural fingerprints can detect specific patterns in its input signals and encode these stimuli in its collective dynamics. The processing and encoding ability of the network is related to the competition established between the spontaneous intrinsic activity and/or the activity evoked by the arrival of external stimuli. This competition may change from winner-take-all to winnerless regimes. When the level of spontaneous intrinsic activity is too high, this wins the competition and external stimuli do not propagate through the network, which avoids the generation of spatio-temporal patterns. This provides a “reset” mechanism to the network. Only by increasing the level of spontaneous intrinsic activity, the network stops processing and the previously detected stimuli almost instantaneously disappear from the network. The coexistence of stimuli in the network is related to the different clusterization and coherence of the transient spatio-temporal activity generated in response to external input. When a winner-take-all competition occurs between the spatio-temporal patterns of activity evoked by different stimuli, one stimulus prevails over the others and only its corresponding patterns travel through the network (globally or locally). On the other hand, when a winnerless competition emerges in the network, the total encoding capacity of the network is alternately distributed among different stimuli. The main factors governing the mode of competition are the level of spontaneous activity and the permeability to external stimuli. Both are driven by intraunit parameters (*p_e_* and *p_r_*, respectively). However, changes in the collective dynamics are also observed as a function of the network topology. The presence of random connections has a similar effect to increase the permeability to external stimuli. These connections facilitate the detection of specific external stimulus in the sense that they increase the stimulus propagation velocity and the level of activity related to stimuli. Networks with a more regular topology (connections with close neighbors) usually benefit the competition between external stimuli. This implies that these networks are able to encode a larger number of stimuli simultaneously.

When the stimulation is over, the same competition regimes emerge between the coexisting spatio-temporal patterns following external stimuli. The only difference is that, now, these patterns are not supported by stimulation and, therefore, when a stimulus looses the competition with other stimuli or with the spontaneous intrinsic activity, it completely disappears from the network. In this way, the patterns of activity induced by external stimuli can almost instantaneously disappear from the network when the stimulation is over. This can be a desirable behavior for some systems. However, the more interesting situations are those where the network displays short-term or long-term memory abilities. In short-term memory networks the stimulus induced activity transiently reverberate within the network. Conversely, in long-term memory networks, the stimuli lead the network to a new stable state where the reverberating patterns related to stimuli becomes a network effect. Then, the stimulus persistently survives in the network until a new stimulus arrives. A short-term memory can become a long-term memory only by increasing the neurons' permeability to external stimuli, and vice versa. In the same way, increasing the number of connections to close neighbors benefits short-term memory mechanisms; while increasing the presence of random connections potentiates the long-term memory mechanisms.

The results reported in this paper indicate that information processing based on the identification of specific neural fingerprints can be a plausible and flexible strategy for neural systems. Different complex dynamic regimes are observed in a simple network of neurons that communicate by exchange of neural fingerprints. Intraunit parameters affecting the individual information processing and the network topology can tune the self-organizing properties of the network. This indicates the great adaptability of the network to different modes of operation and, although not addressed in this paper, the large flexibility to implement both synaptic and subcellular plasticity (Davis, 2006). These results call for more realistic models for the activity of individual neurons, which can introduce a higher information processing capacity in the network.

### Conflict of interest statement

The authors declare that the research was conducted in the absence of any commercial or financial relationships that could be construed as a potential conflict of interest.
